# Identification of a Pharmacological Biomarker for the Bioassay-Based Quality Control of a Thirteen-Component TCM Formula (Lianhua Qingwen) Used in Treating Influenza A Virus (H1N1) Infection

**DOI:** 10.3389/fphar.2020.00746

**Published:** 2020-05-25

**Authors:** Dan Gao, Ming Niu, Shi-zhang Wei, Cong-en Zhang, Yong-feng Zhou, Zheng-wei Yang, Lin Li, Jia-bo Wang, Hai-zhu Zhang, Lan Zhang, Xiao-he Xiao

**Affiliations:** ^1^Department of Pharmacy, Xuanwu Hospital of Capital Medical University, National Clinical Research Center for Geriatric Diseases, Beijing Engineering Research Center for Nervous System Drugs, Beijing Institute for Brain Disorders, Key Laboratory for Neurodegenerative Diseases of Ministry of Education, Beijing, China; ^2^Department of China Military Institute of Chinese Materia, the Fifth Medical Centre, Chinese PLA (People's Liberation Army) General Hospital, Beijing, China; ^3^Department of Pharmacy, the Sixth Medical Center, Chinese PLA (People's Liberation Army) General Hospital, Beijing, China; ^4^College of Pharmacy and Chemistry, Dali University, Dali, China

**Keywords:** traditional Chinese medicine, Lianhua Qingwen capsule, influenza A virus (H1N1), pharmacological biomarker, bioassay, quality control

## Abstract

As chemical analysis for quality control (QC) of traditional Chinese medicine (TCM) formula is difficult to guarantee the effectiveness, a bioassay method that combines QC with evaluation of therapeutic effects has been developed to assess the TCM quality. Here, we chose a thirteen-component TCM formula, Lianhua Qingwen capsule (LHQW), as a representative sample, to explore the pivotal biomarkers for a bioassay and to investigate close association between QC and pharmacological actions. Initially, our results showed that chemical fingerprinting could not effectively distinguish batches of LHQW. Pharmacological experiments indicated that LHQW could treat influenza A virus (H1N1) infection in the H1N1 mouse model, as claimed in clinical trials, by improving pathologic alterations and bodyweight loss, and decreasing virus replication, lung lesions and inflammation. Furthermore, by using serum metabolomics analysis, we identified two important metabolites, prostaglandin F_2α_ and arachidonic acid, and their metabolic pathway, arachidonic acid metabolism, as vital indicators of LHQW in treatment of influenza. Subsequently, macrophages transcriptomics highlighted the prominent role of cyclooxygenase-2 (COX-2) as the major rate-limiting enzyme in the arachidonic acid metabolism pathway. Finally, COX-2 was validated by *in vivo* gene expression and *in vitro* enzymatic activity with 43 batches of LHQW as a viable pharmacological biomarker for the establishment of bioassay-based QC. Our study provides systematic methodology in the pharmacological biomarker exploration for establishing the bioassay-based QC of LHQW or other TCM formulas relating to their pharmacological activities and mechanism.

## Introduction

Traditional Chinese medicine (TCM) formula, as the primary form of TCM in the clinic, plays a vital role in the modernization of TCM (Lin et al., 2018). However, TCM formula usually contains a variety of herbs and can even include mineral and animal components, and consequently, the chemical compositions are far more complicated than that of a single herb (Shi et al., 2015; Zhang J. et al., 2018). The qualitative and quantitative determination of chemical constituents is challenging for the objective evaluation of the quality of TCM formula. Pharmaceutical researchers have realized that several o the well-known analysis methods such as microscopic inspection, active components determination, DNA fingerprinting, and chromatographic fingerprinting does not effectively reflect the consistency of TCM quality and efficacy (Xiong et al., 2014). However, biomarkers as the relevant targets based on the drug efficacy can play a robust role in quality management (Wu et al., 2018). The *Botanical Drug Development Guidance for Industry* issued by FDA in 2016 states that “because of the heterogeneous nature of a botanical drug and possible uncertainty about its active constituent, the technical challenges for quality control are to determine a botanical drug's identity and ensure its consistency of strength. It may require additional measures such as biological assays on the effect of variations, and a biological assay that reflects the drug's known or intended mechanism of action is preferred” (Food and Drug Administration, 2016). Xiao demonstrated that bioassays based on the biological effects of TCM using animals, isolated tissues and organs, microorganisms or cells has been an effective way to improve the quality control (QC) of TCM (Xiao et al., 2014). Therefore, it is of value to explore the pivotal biomarkers for bioassays, and thereby, engage a close association between QC and pharmacological activity that has the potential for ensuring the consistency and clinical efficacy of TCM formulas.

Metabolomics is a burgeoning “omics” field following genomics, proteomics, and transcriptomics and is a powerful approach in biomarker discovery and metabolic pathway exploration that provides a deeper understanding of molecular mechanisms (Griffin et al., 2011); as Prof. Lasley, University of California, Davis, said: “genomics and proteomics tell you what might happen, while metabolomics tells you what did happen” (Bjerrum et al., 2008). Transcriptomics, also called gene expression profiling, is capable of surveying genome-wide transcription to screen candidate target genes and pathways and has been successfully applied to uncover potential biomarkers or yield further insight into disease treatment (Seban et al., 2019). Taken together, the integration of transcriptomics and metabolomics reveal whole genetic and metabolic profile changes of living systems, which is coincident with the characteristics of TCM theory being based on multi-components that work as a holistic system in the treatment of disease (Liu and Locasale, 2017). Thus, an omics-based approach can play a significant role in exploring the mechanism of drug action for complex preparations similar to TCM formulas, which may help to find appropriate biomarkers for bioassay methodology.

Influenza is an acute viral respiratory infection, whose main complications include pneumonia (Self et al., 2014; Sengupta et al., 2019), respiratory failure (Zhang Y. et al., 2018), heart damage (Christiansen et al., 2019), and nervous system damage (Popescu et al., 2017). The influenza A virus can mutate and spread rapidly and can quickly become prevalent, requiring both the health authorities and the public to maintain a continual alert for the treatment and prevention of influenza (Long et al., 2019). From the perspective of TCM theory, the function of Lianhua Qingwen capsule (LHQW) in the body is to release stagnated lung-qi, clear heat, and detoxify, and LHQW has been shown to have a dramatic effect on influenza and acute upper and lower respiratory tract infection (Duan et al., 2011; Dong et al., 2014; Ding et al., 2017). LHQW is a large prescription, composed of 13 kinds of TCM, and has successfully entered a phase II clinical trial in the USA, which means that QC for LHQW is being developed to achieve internationalization (National Pharmacopoeia Committee, 2015). Nevertheless, due to the complexity of chemical constituents and ambiguity of the mechanism of action (Jia et al., 2015; Wang et al., 2016), it is difficult to select the appropriate quality evaluation index and QC method; therefore we explored viable biomarkers for a LHQW bioassay that can supplement QC methodologies and relate to the pharmacological activity and mechanism of action.

Here we explored serum metabolomics to obtain metabolic biomarkers and pathways in the treatment of influenza-like symptoms with LHQW based on the H1N1 influenza virus mouse model. Transcriptomics was then used to select and confirm the potential biomarkers. Finally, the pharmacological biomarker that was suitable to be applied as a bioassay for the QC of LHQW was validated in different batches of LHQW. This study provides a systematic approach for the exploration of pharmacological biomarkers for anti-influenza treatment with LHQW and aims to improve the existing QC system by correlating pharmacological activity with the mechanism of action. Additionally, we sought to provide insight into establishing effective QC systems for other TCM formulas or medicines with heterogeneous composition and uncertainty as to their active components.

## Material and Method

### Chemicals and Reagents

Lianhua Qingwen capsules (LHQW) were commercially purchased from Shijiazhuang Yiling Pharmaceutical Co., Ltd (Shijiazhuang, China); hematoxylin and eosin (HE) were purchased from Zhuhai Beso Biotechnology Co., Ltd. (Zhuhai, China); chloroform, isopropanol and anhydrous ethanol were purchased from Sinopharm Chemical Reagent Co., Ltd (Shanghai, China); lipopolysaccharide (LPS) and penicillin/streptomycin were obtained from Sigma-Aldrich (St. Louis, MO, USA); interferon-gamma (IFN-γ) was purchased from PeproTech (Rocky Hill, USA); high glucose Dulbecco's Modified Eagle's medium (DMEM) was purchased from HyClone (Logan, Utah, USA); fetal bovine serum and 0.25% trypsin were purchased from Gibco (Grand Island, New York, USA); standards of chlorogenic acid, caffeic acid, isochlorogenic acid B, isochlorogenic acid C, phillyrin, forsythiaside A and rutin were purchased from the Chengdu Pufei De Biotech., Ltd (Chengdu, China) and the purity of all these compounds was higher than 98.0%; HPLC-grade methanol and acetonitrile were purchased from Merck (Darmstadt, Germany). Deionized water was prepared using a Milli-Q water purification system Millipore (Bedford, MA, USA). Other chemicals were analytical grade, and their purity was above 99.5%. cyclooxygenase-2 (COX-2) inhibitor screening kit was purchased from Beyotime biotechnology Co., Ltd (Shanghai, China).

### LHQW Samples Preparation and Multicomponent Quantification by HPLC

As China Pharmacopoeia records, Lianhua Qingwen Capsule (LHQW) consists of Forsythiae Fructus [*Forsythia suspensa* (Thunb.) Vahl], Lonicerae Japonicae Flos (*Lonicera japonica* Thunb.), Ephedrae Herba (honey-fried) (*Ephedra sinica Stapf*, *Ephedra intermedia* Schrenk et C. A. Mey. or *Ephedra equisetina* Bge.), Armeniacae Semen Amarum (stir-baked) [*Prunus armeniaca* L. var. *ansu* Maxim., *Prunus sibirica* L., *Prunus mandshurica* (Maxim.) Koehne or *Prunus armeniaca* L.], Gypsum Fibrosum, Isatidis Radix (*Isatis indigotica* Fort.), Dryopteris Crassirhizomatis Rhizoma (*Dryopteris crassirhizoma* Nakai), Houttuyniae Herba (*Houttuynia cordata* Thunb.), Pogostemonis Herba [*Pogostemon cablin* (Blanco) Benth], Rhei Radix et Rhizoma (*Rheum palmatum* L., *Rheum tanguticum* Maxim, ex Balf. or *Rheum officinale* Baill), Rhodiolae Crenulatae Radix et Rhizoma [*Rhodiola crenulate* (Hook. f. et Thoms.) H. Ohba], menthol and Glycyrrhizae Radix et Rhizoma (Glycyrrhiza uralensis Fisch., *Glycyrrhiza inflata* Bat. or *Glycyrrhiza glabra* L.), and the excipient is starch. These can be made into 1,000 capsules, 0.35 g per capsule (National Pharmacopoeia Committee, 2015). The granules from 40 batches of LHQW sold in the market were ground into fine powder in a mortar. Adding 20 ml of 60% methanol to 0.35 g of powder for ultrasonic treatment in 40 min (power: 250 W; frequency: 40 KHz), the extract was filtrated with 0.22 μm microporous filter membrane and collected as the samples 1–40 (S1–S40). It was worth mentioning that S40 was randomly selected out as the object for the following pharmacological effects. Besides, the granule from three batches of LHQW for special treatment including high temperature (60 ℃), high humidity (RH 95%) and light intensity (4,500 lx) for 20 days in the laboratory were the samples 41–43 (S41–S43) respectively. The content of main components of every sample was determined by High Performance Liquid Chromatography (HPLC), and chemical fingerprints of different batches of LHQW were established (**Supplementary Figure S1, Table S1**). Entire methods and any associated operational details are available in the **Supplementary Materials**.

### Virus Strains and Cells

Influenza A virus H1N1 (A/PR/8/34) purchased from American type culture collection, was propagated in the allantoic sac of 9–11-day old embryonated chicken eggs for three days at 37 ℃. Allantoic fluid was harvested, aliquoted, and stored at −80°C for further use. The LD_50_ for the H1N1 virus in mice was calculated using the Reed–Muench method. The cells of Madin Darby Canine Kidney (MDCK) and mouse macrophages RAW264.7 were obtained from the Union Cell Bank of the Chinese Academy of Sciences.

### Cell Culture

RAW264.7 cells were grown in high glucose Dulbecco's Modified Eagle's medium (DMEM) containing 10% (v/v) fetal bovine serum, 100 U/ml penicillin and 100 µg/ml streptomycin, in a humidified 5% CO2 atmosphere at 37°C. For treatment, cells at about 70–80% confluence were divided into four groups including the control, the model, the control + LHQW (C + LHQW) and the model + LHQW (M + LHQW). The model group was given LPS with a final concentration of 1 μg/ml and IFN-gamma with a final concentration of 0.2 μg/ml. C + LHQW and M + LHQW groups were co-treated with 400 μg/ml LHQW cultured for 24 h.

### Transcriptomics of Macrophages

Twelve samples from the above four groups (three replications as a group) were sequenced at Novogene Bioinformatics Technology Co., Ltd (Beijing, China) using RNA-seq technology. Total RNA of macrophages was isolated using Trizol reagent (Invitrogen, USA) according to the manufacturer's instructions. The RNA was quantified with a Nanodrop spectrophotometer (IMPLEN, CA, USA) and RNA integrity was assessed with an Agilent 2100 Bioanalyzer (Agilent Technologies, CA, USA) before the library preparation. A total amount of 3 µg RNA per sample was used as input material for the RNA sample preparations. Sequencing libraries were generated using NEBNext^®^ Ultra™ RNA Library Prep Kit for Illumina^®^ (NEB, USA) following the manufacturer's recommendations. The clustering of the index-coded samples was performed on a cBot Cluster Generation System using TruSeq PE Cluster Kit v3-cBot-HS (Illumia) according to the manufacturer's instructions. After cluster generation, the library preparations were sequenced on an Illumina Novaseq platform and 150 bp paired-end reads were generated. Differential expression analysis was performed using the DESeq2 R package (1.16.1). Genes with an adjusted *P*-value <0.05 and a fold-change ≥2 were assigned as differentially expressed. Gene Ontology (GO) enrichment analysis of differentially expressed genes was implemented by the clusterProfiler R package to screen significantly enriched (*P*-value <0.05), and then Kyoto Encyclopedia of Genes and Genomes (KEGG) was used for pathway enrichment analysis. The results of RNAseq were submitted to the SRA database, which has been successfully processed and the SRA accession was PRJNA602376.

### Animal Experiments

BALB/c mice, aged 6–8 weeks and weighing 18–20 g, were purchased from the Laboratory Animal Center of the Academy of Military Medical Sciences (License No. SCXK-(military) 2012-0004). Animals received food and water ad libitum under standard husbandry conditions (22 ± 2 °C temperature, 60–80% relative humidity and 12 h photoperiod) in the fifth medical center of the Chinese People's Liberation Army General Hospital and acclimated one week for experiments. For the determination of half lethal dose (LD_50_), 50 BALB/c mice were randomly divided into five groups. The viruses were serially diluted as 10^−2^, 10^−3^, 10^−4^, 10^−5^, 10^−6^ with sterilized phosphate-buffered saline (PBS). After mild anesthesia with ether, mice were infected by nose drop with 50 μl dilution. The survival and death of mice infected with different dilution viruses were recorded for 15 days. LD_50_ was calculated by Reed–Muench method. Afterwards, mice were randomly divided into five groups: control group, model group, oseltamivir positive group, LHQW groups consisting of low and high doses. Mice of oseltamivir group and LHQW groups were treated with 60 mg/kg/day oseltamivir or LHQW 650 mg/kg/day and 1,300 mg/kg/day respectively twice per day (8 h between doses) for five consecutive days (Ding et al., 2017), as well as control and model group were administered with the equivalent vehicle by gavage. Notably, 2 h after the first administration, mice in the model group, oseltamivir group and LHQW groups were in mild anesthesia and inoculated intranasally with LD_50_ ×2 of H1N1 in 50 µl PBS. At the same time, mice of the control group received the same volume of PBS intranasally. The bodyweight of the mouse in each group was recorded daily from the first administration. All animal experiments were repeated three times at least and approved by the Committee on the Ethics of Animal Experiments of the fifth medical center of the Chinese People's Liberation Army General Hospital. (Approval No.: IACUC-2015-011).

### Lung Index, Virus Titer and Histology

Two hours after administration on the fifth day of virus infection, seven mice from each group were euthanized. The whole lung tissue was harvested from the mice, washed twice with saline, dried with filter paper, and weighed. The lung index (LI) was calculated with the formula: LI = [(lung weight/g)/(bodyweight/g)] × 100%. Then three mice lungs selected randomly from sacrificial mice were ground in DMEM medium on ice, and the grinding fluid was centrifuged at 12,000 rpm/min for 10 min. Supernatant was 10-fold serially diluted and titrated in 96-well culture plates of MDCK cells. The titers were calculated using the Reed–Muench method and were expressed as lgTCID_50_/g lung tissue. The same portion of left lung tissue for histopathological examination were fixed with neutral formalin solution for 24 h, embedded in paraffin, sectioned to a thickness of approximately 5 µm, and stained with hematoxylin and eosin (HE).

### Serum Preparation and UPLC-MS Analysis

The blood was taken from ten mice eyeball after last oral administration 2 h later, and centrifuged subsequently for 10 min at 5,000 rpm at 4 °C. Supernatant was pipetted into the tube and preserved at −80 °C for further use. UPLC-MS analysis was described referring to the author's previously published article (Gao et al., 2016). Briefly, samples were extracted by three volumes of precooled (−20 °C) acetonitrile, vortex-mixed, centrifuged subsequently for 30 min at 10,000 rpm at 4 °C. Each supernatant was carefully separate into vials and filtered by a 0.22 μm microfiltration membrane for metabolomic analysis. Metabolomics was performed on Waters Xevo G2-XS QTOF/MS. Chromatography and mass spectrometry conditions were described in the **Supplementary Information** (**Supplementary Table S3**).

### Identification of the Metabolites and Metabolic Pathways Analysis

Methods of metabolites screen and metabolic pathway enrichment were almost the same as described in the previous article (Gao et al., 2016). Endogenous metabolites that contribute to the classification were found by variable importance in the projection (VIP) values. Only VIP values >1 were selected and used for further data analysis. Concerning the selection of potential biomarkers and identification of their molecular formulas, the significant differences should be satisfied with the conditions of *P-*value <0.05 and fold changes >2 calculated by the MetaboAnalyst 3.0 software. The ion spectrum was matched with the structure message of metabolites acquired from available biochemical databases, such as METLIN (http://www.metlin.scipps.edu/), HMDB (http://www.hmdb.ca/) and the Massbank database (http://www.massbank.jp/). Finally, the pathway analysis of potential biomarkers was performed with MetaboAnalyst 3.0 (http://www.metaboanalyst.ca/) based on the pathway library of mouse to identify the metabolic pathways disturbed by the administration of drugs.

### RT-qPCR for the Validation of Significant Changed Candidate Gene

To verify the expression of selected genes *in vivo*, lung tissues (100 mg) from three times repeated animals experiments were homogenized, and total RNA was prepared according to the method mentioned above in “Transcriptomics of macrophage cell lines”. One milligram of total RNA was reverse-transcribed to cDNA using RevertAid™ First Strand cDNA Synthesis Kit (Thermo Scientific, USA) following the manufacturer's protocol. The cDNAs were then used as templates in cDNA Synthesis SuperMix (Novoprotein, China) with a ABI 7500 Real-Time PCR System and 7500 System Software (Applied Biosystems, Alameda, CA, USA) to analyze the gene expression with the cycling program 40 cycles of 95 °C for 15 s, 60 °C for 1 min, and the melting curves were analyzed at 60–95 °C. Glycer-aldehyde 3-phosphate dehydrogenase (GAPDH) was used as an internal reference and each RT-qPCR analysis was performed in triplicate. Primer sequences were designed using Applied Biosystems as below: GAPDH forward primer: 5'-GTCCTCAGTGTAGCCCAAGAT-3', GAPDH reverse primer: 5'-CAATGTGTCCGTCGTGGATCT-3'; COX-2 forward primer: 5'-CCCTGCTGGTGGAAAAGCCTGGTCC-3', COX-2 reverse primer: 5'-TACTGTAGGGTTAATGTCATCTAG-3'. The comparative CT method (2^−ΔΔCT^) was used to calculate the relative changes in target gene expression.

### Statistics

All results were expressed as the mean ± standard deviation (S.D.). Data were analyzed by one-way ANOVA using the SPSS20.0 statistical software (IBM Corporation, Armonk, NY, USA). The difference was considered statistically significant when *P* ≤0.05, and very significant when *P* ≤ 0.01 and *P* ≤ 0.001.

## Results

### High Similarity of Chemical Fingerprint Among 43 Samples of LHQW

Chemical fingerprinting has been recommended as a reliable methodology for the quality evaluation of herbal medicines (Goodarzi et al., 2013). Here, the main components of LHQW included chlorogenic acid, caffeic acid, isochlorogenic acid B, isochlorogenic acid C, phillyrin, forsythiaside A, and rutin were determined by HPLC according to the Chinese Pharmacopoeia (2015). The results showed that the content of each component varied between different samples (**Supplementary Table S2**). Moreover, the chemical fingerprint of each sample was established (**Supplementary Figure S2**) and similarity analysis among them was carried out *via* the Similarity Evaluation System for Chromatographic Fingerprint of Traditional Chinese Medicine (Version 2012A, SES software). Compared to the reference chromatogram generated with average data, the similarity values of all 43 samples were close to 0.85, which indicates minimal difference among samples, including three denatured samples, S41, S42, and S43 (**Table 1**).

**Table 1:** Similarity analysis of chemical fingerprint from different batches of LHQW.

Samples	Similarity	Samples	Similarity	Samples	Similarity	Samples	Similarity
S01	0.904	S12	0.931	S23	0.921	S34	0.914
S02	0.882	S13	0.943	S24	0.874	S35	0.898
S03	0.866	S14	0.949	S25	0.903	S36	0.905
S04	0.852	S15	0.877	S26	0.942	S37	0.901
S05	0.874	S16	0.869	S27	0.929	S38	0.927
S06	0.915	S17	0.935	S28	0.858	S39	0.896
S07	0.996	S18	0.872	S29	0.873	S40	1.000
S08	0.884	S19	0.935	S30	0.916	S41	0.870
S09	0.876	S20	0.858	S31	0.883	S42	0.905
S10	0.848	S21	0.908	S32	0.885	S43	0.856
S11	0.912	S22	0.878	S33	0.883		

### LHQW Improves Influenza-Like Symptoms

#### Phenotypic Changes of Mice

**Figure 1:**
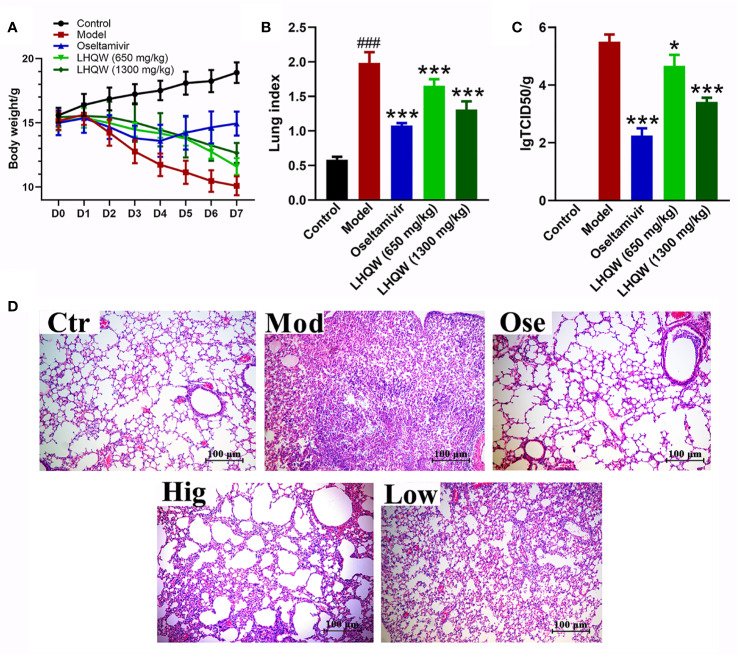
LHQW alleviated H1N1-induced clinical signs in mice. **(A)** Bodyweight changes of mice infected by the HIN1 influenza virus. Mice were infected H1N1 (2 × LD_50_ per mouse) 2 h after the first administration and intragastrically administered twice per day (8-hour interval) for five consecutive days. The bodyweight of mouse was recorded daily and D0 weight was recorded before the day of first administration at the same time. **(B)** BALB/c mice were infected with the H1N1 virus (2×LD_50_ per mouse). At the fifth day of infection, seven mice were euthanized in each group. The mean lung index of each group was determined as lung weight/the final body weight. **(C)** Viral titers were determined by Reed–Muench method. Data are represented as the mean ± standard deviation (S.D.), *n* =7. ^###^*p <* 0.001 vs control group; *p < 0.05, ***p < 0.001 vs model group for one-way ANOVA. **(D)** Lung histology sections stained with hematoxylin and eosin (H&E stained, ×200 magnification) at the fifth-day post-infection. Ctr: the control group; Mod: the model group; Ose: the positive drug oseltamivir group; Hig: high dose of LHQW group (1,300 mg/kg/day); Low: low dose of LHQW group (650 mg/kg/day group).

Mice infected with H1N1 influenza virus became visibly sluggish while their hair appeared to bristle. The bodyweight in the model and drug groups began to decrease on the second day after infection as compared with the control group (not infected), eventually reaching 33% of the original body weight in mice without treatment (**Figure 1A**). With treatment using the anti-influenza drug oseltamivir, the mice showed minor signs of sluggishness and hair-bristle, and the most measurable weight loss was reduced by approximately 9%, after which bodyweight began to rise from the fifth day after falling (**Figure 1**). When treated with LHQW at low (650 mg/kg/d) or high (1,300 mg/kg/d) dose by gavage, the above pathologic phenomena of sluggishness and hair-bristle were moderately improved. Although weight loss also continued at the fifth day after infection, the maximum loss was still less than 20% of the initial body weight (**Figure 1A**); the high dose of LHQW also ameliorated these changes of mice in body weight and hair more effectively than the low dose.

#### LHQW Attenuates Lung Index and Viral Load

Lung index refers to the percentage of lung weight to body weight and can indicate the severity of pneumonia. Influenza virus infection causes viral pneumonia in mice, and consequently, inflammation exudation increases lung weight (Wei et al., 2018). **Figure 1B** demonstrates that the mean lung index of the model group significantly increased compared with the control group on the fifth day of infection (*p <* 0.01). However, treatment with oseltamivir effectively reduced lung index compared to the model group. Treatment with LHQW at low and high dose also significantly decreased indices (*p <* 0.01), and a high dose (1,300 mg/kg/d) of LHQW was more effective than low. Virus titers in the lung were determined to evaluate the effect of drugs on local replication of the H1N1 virus. Treatment with oseltamivir or LHQW could significantly attenuate the titer of the H1N1 virus compared to the model group (*p <* 0.01), and the average value of virus titer were 2.25 ± 0.25, 3.41 ± 0.14, and 4.67 ± 0.38, respectively (**Figure 1C**); there was no virus infection detected in the control group. These results indicate that both low and high doses of LHQW could effectively inhibit the replication of the influenza virus in mice, while the high dose had a greater suppressive effect.

#### LHQW Prevents Lung Inflammation by Histopathological Evaluation

Pathological examination (**Figure 1D****-**Ctr) showed that the lung alveolar structure was clear with integral morphology and size the same as the control and with no inflammatory cells observed. By contrast, lungs from the mice of the model group had severe pneumonia-like symptoms. Structures of alveoli, alveolar wall, and alveolar septum show significant damage, such as thickening of alveolar walls, increase of lung interstitium, and decrease of alveolar space. Significant inflammatory cell infiltration and alveolar exudates were also observed (**Figure 1D****-**Mod). Following treatment with oseltamivir, the pathological damage showed an evident recovery comparative to the control (**Figure 1D****-**Ose). A high dose of LHQW produced clear improvements in structures of alveoli, alveolar wall, and alveolar septum, and although the lungs still showed inflammatory infiltration, this was much improved in comparison with the model group (**Figure 1D****-**Hig). The low dose of LHQW did not improve histological appearances of pneumonia-like symptoms as clearly as the high dose did, but the lesions were less severe than those in the model group (**Figure 1D****-**Low).

### Serum Metabolomics Analysis of Essential Metabolites and Metabolic Pathway

#### Metabolic Variation and Pharmacological Biomarker Identification

Here we examined the serum of mice from control, model, and high dose of LHQW. Based on the changes of endogenous metabolites, the differences in metabolic expression profiles among groups were characterized, and specific biomarkers were screened. UPLC-QTOF/MS in positive and negative ESI modes were applied to acquire the metabolic profile of serum samples and chromatographic peaks that represented small molecular weight metabolites. The retention time, peak intensity, and exact mass was imported into the Masslynx™ software to determine molecular formulas by accurate mass and isotope matching for multiple statistical analyses. Both principal component analysis (PCA) and orthogonal partial least squares discriminant analysis (OPLS-DA) were used to analyze differences among groups due to their ability to manage high-noise, collinear, highly multivariate, and possibly incomplete data.

**Figure 2:**
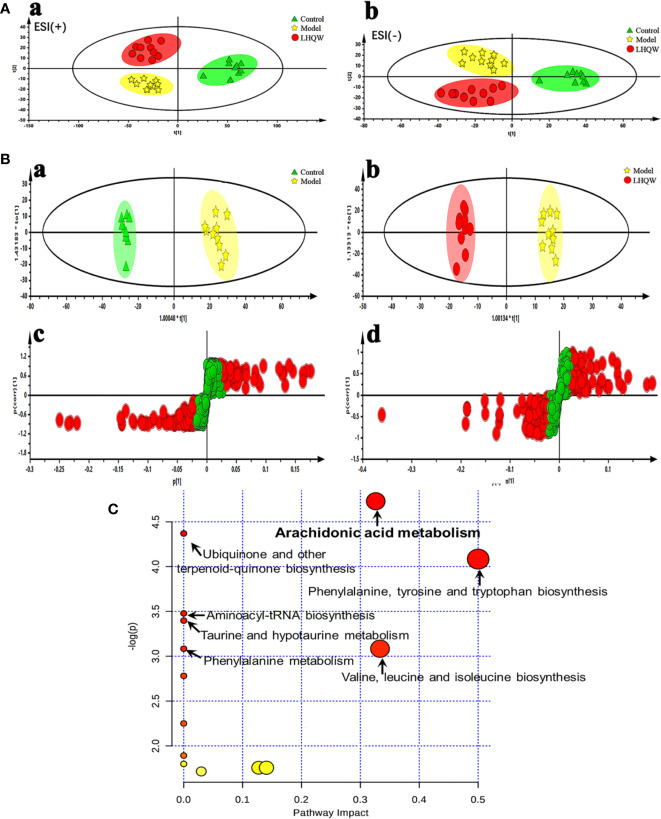
Serum metabolomics analysis of changes in metabolic profiles among control, model and high dose of LHQW groups. Mice were randomly divided into three groups: control group, model group and high dose LHQW group (1,300 mg/kg/day) (*n* = 10). Model and LHQW groups were infected with H1N1 (2 × LD_50_ per mouse) 2 h after the first administration and intragastrically administered twice per day (8-hour interval) for five consecutive days. The blood was taken from eyeball after the last oral administration 2 h later, and centrifuged subsequently to obtain supernatant for use. **(A)** PCA scores plot of comparing control, model and LHQW groups in positive ESI modes (a) and negative ESI modes (b). **(B)** The OPLS-DA score plots and S-plots generated from the OPLS-DA of the QTOF/MS data from control, model and LHQW groups in the ESI^−^ mode. OPLS-DA score plots were the pair-wise comparisons between the control and the model (a) as well as the model and LHQW (b). S-plot of the OPLS-DA model was for the control and the model (c) and the model and LHQW (d), whose axes plotted in the S-plot from the predictive component are p1 vs. p(corr)1, representing the magnitude (modeled covariation) and reliability (modeled correlation) respectively. **(C)** Summary of pathway analysis with MetaboAnalyst 3.0 based on the differential metabolites.

Unsupervised PCA analyses was performed to visualize inter-group metabolic differences, in which each shape represents an individual sample, and the distance between shapes reflects the degree of their metabolic differences. The score plot of the PCA with the metabolic data of ESI^+^ mode and ESI^−^ mode showed evident separation between the control and model groups, indicating that the metabolic profiles of influenza virus H1N1 infected animals had changed substantially. The model and the LHQW group were divided into two clusters, which suggests that LHQW had a major influence on the metabolic behavior of mice infected with H1N1 (**Figure 2A**).

Additionally, OPLS-DA was applied to amplify the differences between groups and explore potential biomarkers related to the effect of LHQW for influenza-based therapy. The quality of the PCA and OPLS-DA models are summarized in **Supplementary Table S4**. Parameters including R_2_Y (cum) and Q2 (cum) in pair-wise groups larger than 0.5, suggested that each model was robust with good fitness and prediction. Score plots of the supervised OPLS-DA showed distinct separation between the control and the model group and the model and the LHQW group both in negative and positive ion modes (**Figure 2B-a,b** and **Supplementary Figure S3A, B**), which indicates that biochemical perturbation in the serum occurred conspicuously in the model group; LHQW also altered this perturbation to some extent. In the corresponding S-plots (**Figure 2B-c, d** and **Supplementary Figure S3C, D**), the variables farthest from the center were responsible for the changes of metabolic pattern and separation between groups, and might therefore be regarded as potential biomarkers on the condition that the variable importance in projection (VIP) values were higher than one (VIP >1.0). To reduce the risk of false positives in the pharmacological biomarker screening, variables with |p(corr)| ≥0.5 were selected to ensure they correlated most with the OPLS-DA discriminant scores between groups.

Co-metabolites that changed significantly (*p <* 0.05) in the model group compared with the control group as well as the LHQW group were selected as candidate biomarkers. The criterion of a 1.5-fold change was restricted as the feature of an average normalized intensity difference. Metabolites in the ESI^+^ and ESI^−^ mode analyses were combined to perform the further identification of their molecular formulas. All biomarkers were preliminarily identified with the accurate mass charge ratio by the online METLIN database (http://www.metlin.scipps.edu/), and then targeted MS/MS analysis was applied to determine the ion structures for identification of the metabolites. Using the method described above, a total of six potential biomarkers, including three metabolites, L-tyrosine, L-valine, and taurocholic acid, from the ESI^+^ analysis, and three metabolites, L-ornithine, prostaglandin F_2α_, (PGF_2α_), and arachidonic acid (AA) from the ESI^−^ analysis were identified and listed in **Supplementary Table S5**. The results revealed that some metabolites, such as PGF_2α_ and AA, were consistent with the improvements observed in clinical signs and histological findings of LHQW for lung inflammation.

#### **Biological** Pathway Anal**ysis of** Identified Pharmacological Bioma**rkers**

MetaboAnalysis, a free network platform for comprehensive analysis of quantitative metabolome data, was used to analyze the metabolic pathways based on the above six differential metabolites. As shown in **Figure 2C**, we targeted seven potential metabolic pathways related to LHQW treatment of influenza (**Supplementary Table S6**), with one of the most critical pathways being arachidonic acid metabolism

### Pharmacological Biomarker Orientation for Bioassay of LHQW by Cell Transcriptomics

**Figure 3:**
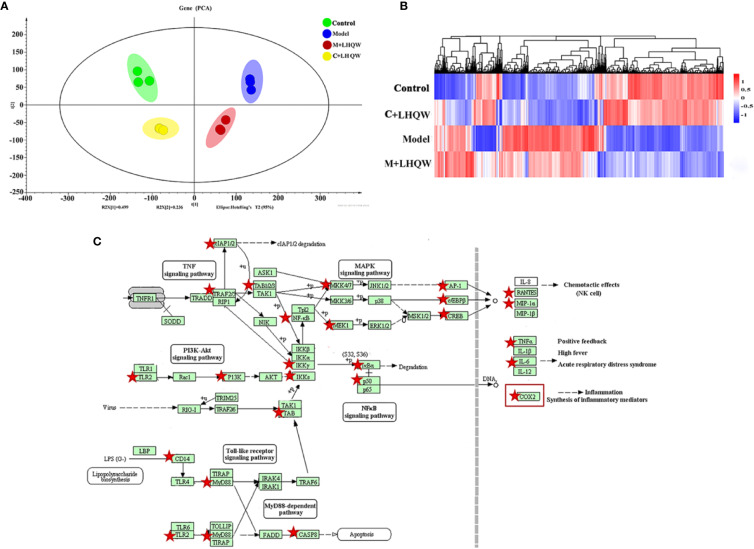
The analysis of differential expression genes and pathways by cell transcriptomics as LHQW treatment. RAW264.7 cells were divided into four groups including the control, the model, the control + LHQW (C + LHQW) and the model + LHQW (M + LHQW). Model was made with1 μg/ml LPS and 0.2 μg/ml IFN-gamma. C + LHQW and M + LHQW groups were co-treated with 400 μg/ml LHQW cultured for 24 h. Total RNA from cells was isolated and sequenced. **(A)** PCA scores plot of comparing control, model, C + LHQW and M + LHQW groups for cell differential expression genes. **(B)** Heatmap of genes expression showed the variation trend among the four groups. **(C)** Comprehensive pathway analysis of differential expression genes by cell transcriptomics. Red stars represented genes closely related to the effect of LHQW anti-inflammation, and COX-2 as one of the most downstream targets coincide with the above metabolomics results.

Based on the results above, LHQW significantly reduced the influenza-induced lung inflammation in mice. Next, RAW264.7 mouse macrophages were stimulated by lipopolysaccharide (LPS) combined with interferon gamma (IFN-γ) and were used as the inflammatory cell model to assess the response to LHQW treatment (Li et al., 2019). Transcriptomics was employed to screen the gene indicators related to the anti-inflammatory mechanism of LHQW. A genome-wide analysis showed a number of differential expression genes (DEGs) from three pairs of analyses, including control vs. control + LHQW (C + LHQW), control vs. model, and model vs. model + LHQW (M + LHQW). Standardized data of DEGs were imported to the SIMCA-P-13 software for PCA to observe the diversity among samples. The control group, model group, C + LHQW group, and M + LHQW group were differentiated, which indicated that the expression profiles of RAW264.7 cells in normal and inflammation stimulation were significantly changed after LHQW intervention (**Figure 3A**). More explicitly, the heatmap of expression genes indicated there was a major difference between the control group and the model group according to the degree of color variations, which improved after LHQW administration (**Figure 3B**).

We then undertook KEGG pathway analyses of all screened DEGs. Several pathways were inflammation-related, including NF-kappa B signaling pathway, toll-like receptor signaling pathway, TNF signaling pathway, influenza A, NOD-like receptor signaling pathway (data not shown). Integrating these pathways and the changes of DEGs, 26 genes, which are represented by red stars, closely related to the anti-inflammatory effect of LHQW were screened (**Figure 3C**). In particular, cyclooxygenase-2 (COX-2) was prominent in the metabolomics results of LHQW for the treatment of influenza-like symptoms in the pathway of arachidonic acid metabolism.

### Validation of the Potential Biomarker for LHQW Quality Control

**Figure 4:**
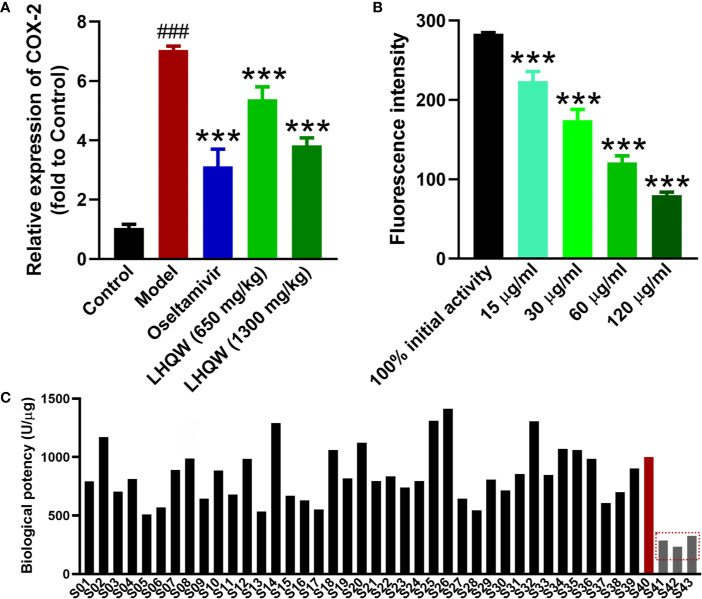
Validation of COX-2 as the potential biomarker for LHQW quality control. **(A)** RT-qPCR was used to validate the COX-2 mRNA expression in lungs of each group as described in **Figure 1**. RNA of the internal control gene GAPDH was used to calculate the relative expression of COX-2 according to the 2^−ΔΔCt^ mathematic method (*n*  = 7). ^###^*p <* 0.001 vs control group; ***p < 0.001 vs model group for one-way ANOVA. **(B)** The COX-2 activity inhibition of LHQW *in vitro* using the COX-2 inhibitor screening kit (*n*  = 3), ***p <0.001 vs the100% initial activity for one-way ANOVA. Data are represented as the mean ± standard deviation (S.D.). **(C)** The biological potency of 43 LHQW samples was measured using the COX-2 inhibitor screening kit. Forty three samples including 40 commercial samples and three samples suffered destructive treatment such as high temperature (60°C), high humidity (RH 95%) and light intensity (4,500 lx) for 20 days in the laboratory as the S41–S43 respectively. The biological potency of LHQW S40 was defined as the reference for 1,000 U/μg, and other samples were compared with it to calculate the value.

Based on the metabolomics and transcriptomics analysis, COX-2 was a key target in treating influenza pneumonia with LHQW. To verify these results, RT-qPCR was performed to further investigate *COX-2* expression in mice lungs. The level of *COX-2* mRNA was significantly up-regulated in the mouse model infected by the H1N1 influenza virus compared with the control (**Figure 4A**). Low- and high-doses of LHQW decreased *COX-2* expression in comparison with the model group, while the high dose of LHQW was more effective than the low dose and achieved similar results to treatment with oseltamivir.

Through the above validation, COX-2 was confirmed as a valid target for the quality control and evaluation of LHQW related to clinical efficacy. Cyclooxygenase (COX) is a bifunctional enzyme, also known as prostaglandin-endoperoxide synthase (PTGS). COX-2 is frequently and closely related to the development of inflammation (Meriwether et al., 2019) and tumor development (Kosumi et al., 2019). Therefore, it may be convenient and efficient to use a COX-2 inhibitor screening kit for the biological assessment of LHQW with the COX-2 activity. According to the kit protocol, a series of LHQW concentrations were assessed for inhibition of COX-2 enzyme activity. The result showed the fluorescence intensity in each LHQW group was concentration dependent and significantly decreased compared with the initial activity (**Figure 4B**). This result indicated LHQW had a significant inhibitory effect on COX-2 activity *in vitro* in a dose-dependent manner, and that COX-2 could be used for the biological evaluation of LHQW.

Furthermore, using the COX-2 inhibitor screening kit, we tested the inhibitory effect of the 43 LHQW samples, including 40 commercial samples and three denatured samples. The biological potency of LHQW S40 sample was defined as the reference for 1,000 U/μg, while the other samples were designated as test products. There were clear differences in the biological potency of COX-2 inhibition among batches. The biological potency ranged from 510 U/μg to 1,413 U/μg in the 40 batches of commercial samples, while the denatured samples were significantly lower than that of the commercial samples, suggesting that this method has sufficient sensitivity to distinguish the quality of different LHQW samples (**Figure 4C**).

## Discussion

Lianhua Qingwen Capsule (LHQW) has been widely used for almost 15 years in China as an anti-influenza medication. Indeed, LHQW exhibits multiple pharmacological effects demonstrated in modern research including antiviral, anti-inflammatory, immunomodulatory, and antipyretic actions, which have distinct therapeutic effects on diseases like influenza and pneumonia (Duan et al., 2011; Dong et al., 2014; Zhao et al., 2014; Ding et al., 2017). However, LHQW is a complex prescription and chemical composition analysis is insufficient to distinguish the quality differences needed to guarantee clinical effectiveness. Here we explored viable biomarkers to establish a bioassay associated with the clinical efficacy for LHQW together with the supplementary quality control (QC) methodologies. First, we demonstrated that chemical fingerprinting cannot yet effectively distinguish between multiple samples of LHQW. Alternatively, by combining the pharmacologic effects with omics analysis, we identified COX-2 as a pivotal biomarker that reflected the mechanism of LHQW in the treatment of influenza. Finally, COX-2 was validated *in vitro* and *in vivo* as a feasible pharmacological biomarker, which was demonstrated by successfully distinguishing different batches of LHQW and, in particular, denatured samples. In brief, COX-2 has the potential ability for the QC of LHQW as a bioassay indicator related to the pharmacological activities and mechanism of action.

LHQW contains a number of chemical entities that act on multiple targets, and therefore, it is a major challenge to systematically study the mechanism of action using conventional methods. Thus, in the present study, we applied metabolomics combined with transcriptomics to explore the LHQW related pathways and therapeutic targets. A total of six potential biomarkers, including L-ornithine, prostaglandin F_2α_ (PGF_2α_), arachidonic acid (AA), L-tyrosine, L-valine, and taurocholic acid were selected. KEGG pathway analyses identified seven potential metabolic pathways, a key one being arachidonic acid metabolism. Two potential biomarkers PGF_2α_ and AA, together with arachidonic acid metabolism were consistent with the improvements for lung inflammation observed in pharmacological experiments of LHQW in the treatment of H1N1 influenza-infections. Subsequently, the transcriptomes of mouse macrophages identified 26 differential expression genes (DEGs) and COX-2 as the most prominent downstream target, which coincided with the identification of the arachidonic acid metabolic pathway *via* metabolomic analyses.

The arachidonic acid metabolic network is the main network for producing inflammatory mediators (Sala et al., 2018). There are three critical enzymes, including cyclooxygenase (COX), lipoxygenase (LOX), and cytochrome P450 (CYP450) involved in the regulation of AA metabolism, and a series of potent bioactive substances are produced, such as prostaglandins (PGs), thromboxanes, leukotrienes, and epoxyeicosatrienoic acids (Mesaros and Blair, 2012; Hanna and Hafez, 2018). COX is the rate-limited enzyme for converting AA into PGs, which are an essential class of inflammatory mediators (Sachett and Verli, 2011; Akasaka and Ruan, 2016). In particular, COX-2, an isozyme of COX, is an inducible enzyme that is produced rapidly by stimulation, and then catalyzes the synthesis of PGs relating to the inflammatory response (Johnsen et al., 2004; Akasaka and Ruan, 2016). Research has focused on the specific inhibition of the COX-2 expression to develop anti-inflammatory or pain-relieving drugs (Vardeh et al., 2009; Martelli et al., 2013). In this study, COX-2 was up-regulated in both cell and mouse models, whereas LHQW reduced the levels of COX-2. Thus, we speculate that LHQW primarily acted on the COX-2 target through the arachidonic acid metabolism pathway to treat influenza-induced pneumonia.

TCM formulas have accumulated thousands of years of clinical experience and have attracted worldwide attention for definitive efficacy with few side effects. In contrast, their diverse chemical composition, unclear mechanism of action, and inadequate quality control have brought challenges for their modernization and internationalization. At present, the QC of LHQW is specified in the Pharmacopoeia 2015 edition as each capsule containing no less than 0.17 mg of phillyrin (2015). However, chemical fingerprinting cannot effectively distinguish between different samples of LHQW, especially using an individual index component such as phillyrin, so detection of potential biomarkers may be a sensitive and powerful supplement for the QC of LHQW. Here, COX-2 was selected as a potential biomarker associated with the pharmacological activity of LHQW on influenza pneumonia for quality evaluation, mainly *via* omics technology to highlight the global effects of the treatment. In addition, LHQW has been clinically applied for treating influenza-like symptoms, which are most associated with inflammation, inflammatory cytokines, or inflammatory metabolites; LHQW has also been approved for evaluation in a phase II clinical trial by the FDA. Therefore, our conclusion in selecting COX-2 as one of the evaluating indicators for LHQW is well-founded and viable.

In summary, this study demonstrates that high dosage of LHQW (1,300 mg/kg/day) produced significant improvements in body weight loss, virus replication, and especially lung inflammation, following infection by H1N1 influenza virus. Furthermore, metabolomics integrated with transcriptomics determined that LHQW had a distinct metabolic influence in the treatment of influenza pneumonia, mainly targeting the COX-2 in the arachidonic acid metabolism pathway. Finally, COX-2 was verified in gene expression, enzymatic activity, and different batches of LHQW to be a reliable and viable biomarker, which may be sufficient to establish a biological assay for QC relating to the pharmacology, activity, and mechanism of LHQW; whereas chemical fingerprinting fails to effectively distinguish inferior products from commercial samples of LHQW. However, testing of human samples will be necessary in further research to confirm the critical role of COX-2. In addition, COX-2 testing should be implemented extensively to assess LHQW preparations prepared under different conditions. The study of pharmacological biomarker exploration may provide not only scientific information to further understand the mechanism of LHQW in treating influenza-like symptoms, but through an innovative manner (the “omics” network combined with validation *in vivo* and *in vitro*) may also establish a QC method for LHQW relating to the pharmacology activity and mechanism of action. Here, we used LHQW, a representative TCM formula in the clinic, as the model drug to explore the pharmacological biomarkers for bioassay, which has provided insights into other complex TCM formulas or herbs. This may have considerable theoretical significance and commercial value in ensuring the quality of the TCM preparations in clinical applications.

## Data Availability Statement

The datasets generated for this study can be found in NCBI BioProject (PRJNA602376) https://www.ncbi.nlm.nih.gov/bioproject/PRJNA602376.

## Ethics Statement

The animal study was reviewed and approved by the Committee on the Ethics of Animal Experiments of the fifth medical center of Chinese People's Liberation Army General Hospital. (Approval No.: IACUC-2015-011).

## Author Contributions

DG and MN performed the investigation and wrote the paper. S-ZW and C-EZ participated in the collection and preparation of samples. Y-FZ and Z-WY performed the analyses. LL, J-BW, LZ, H-ZZ, and X-HX designed the study and amended the paper. All authors reviewed and approved the final manuscript.

## Conflict of Interest

The authors declare that the research was conducted in the absence of any commercial or financial relationships that could be construed as a potential conflict of interest.
