# Supplementary material for: Identification of a Pharmacological Biomarker for the Bioassay-Based Quality Control of a Thirteen-Component TCM Formula (Lianhua Qingwen) Used in Treating Influenza A Virus (H1N1) Infection
Source: Front Pharmacol. 2020 May 25;11:746. doi: 10.3389/fphar.2020.00746 (PMC7261828; doi:10.3389/fphar.2020.00746)
Supplement: Supplementary file 1 [file DataSheet_1.docx]

***Supplementary materials for*：****Identification of a Pharmacological Biomarker for the Bioassay-based Quality Control of a thirteen-component TCM formula (Lianhua Qingwen) used in Treating Influenza A Virus (H1N1) Infection**

Dan Gao^1,2‡^, Ming Niu^2‡^, Shi-zhang Wei^2^, Cong-en Zhang^2^, Yong-feng Zhou^2^, Zheng-wei Yang^3^, Lin Li^1^, Jia-bo Wang^2^, Hai-zhu Zhang^2,4*^, Lan Zhang^1*^, Xiao-he Xiao^2*^

1. *Department of Pharmacy, Xuanwu Hospital of Capital Medical University, National Clinical Research Center for Geriatric Diseases, Beijing Engineering Research Center for Nervous System Drugs, Beijing Institute for Brain Disorders, Key Laboratory for Neurodegenerative Diseases of Ministry of Education, Beijing 100053, China*
2. *Department of China Military Institute of Chinese Materia,* *the Fifth Medical Centre, Chinese PLA (People’s Liberation Army) General Hospital, Beijing 100039, China*
3. *Department of Pharmacy, the Sixth Medical Center, Chinese PLA (People’s Liberation Army) General Hospital, Beijing 100048, China*
4. *College of Pharmacy and Chemistry, Dali University, Dali 671000, China*

Corresponding authors: Hai-zhu Zhang, e-mail: [hzningjing@163.com](mailto:hzningjing@163.com); Lan Zhang, e-mail: [lanizhg@126.com](mailto:lanizhg@126.com); Xiao-he Xiao, e-mail: [pharmacy302xxh@126.com](mailto:pharmacy302xxh@126.com), Fax: +86 66933322; +86 66933325, Postal address: the Fifth Medical Centre of Chinese PLA (People’s Liberation Army) General Hospital, No. 100 Xisihuan Beijing 100039, China

‡ These authors contributed equally to this work.

* To whom correspondence should be addressed.

# Multicomponent quantification of Lianhua Qingwen capsules (LHQW) by HPLC

Analyses were performed using the Waters Acquity UPLC system (Waters Technologies, Milford, Massachusetts, USA). Chromatography was carried out at 30ºC on a Waters Acquity HSS T3 column (100 mm × 2.1mm, with 1.7 μm particle size). The mobile phase consisted of (A) methanol and (B) 0.01% (v/v) phosphoric acid water solution. The gradient elution was as follows: 15-20% A from 0 to 8 min, 20-24% A from 8 to 15 min, 24-30% A from 15 to 20 min, 30-34% A from 20 to 25 min, 34-40% A from 24 to 30 min, 40-50% A from 30 to 35 min, 50-60% A from 35 to 40 min at a flow rate of 0.2 mL⋅min^−1^. The signal was monitored at 210 nm.


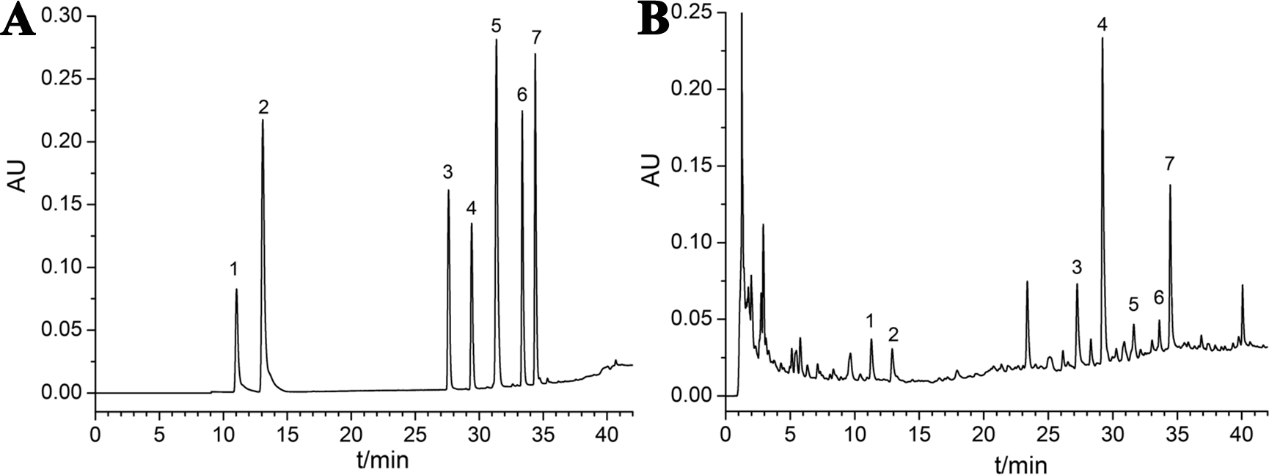


**Figure S1** Multicomponent quantification of the LHQW. (**A**) Chromatogram of chlorogenic acid, caffeic acid, forsythiaside A, isochlorogenic acid B, rutin, isochlorogenic acid C and phillyrin standard mixture. Peak 1 for chlorogenic acid, peak 2 for caffeic acid, peak 3 for forsythiaside A, peak 4 for isochlorogenic acid B, peak 5 for rutin, peak 6 for isochlorogenic acid C and peak 7 for phillyrin. (**B**) HPLC profile of LHQW sample numbered S40.

# The HPLC fingerprint analysis of LHQW

Sample no. 40 (S40) was selected as a representative sample to validate the method for fingerprint analysis. Method precision and reproducibility were evaluated by the analysis of five injections and five injections solution prepared independently from S40. Relative standard deviation (RSD) values of the relative retention time (RRT) and the relative peak area (RPA) for some characteristic peaks, including 7 peaks (Figure S1B, peak no. 1, 2, 3, 4, 5, 6, 7) were calculated. RSDs of RRT and RPA in the precision test (n = 5) were found in the range of 0.13-0.21% and 1.71-5.01% respectively. Reproducibility (n = 5) for both RRT (0.11 - 0.21% RSD) and RPA (1.87 - 4.02% RSD) were acceptable. The stability study of the sample was performed within 24 h (0, 3, 6, 12, 24 h), and stability (RSD) showed less than 0.37% for RRT and 5.64% for RPA, indicating that the sample was stable for 24 h. In total, the RSDs of RTT were less than 0.37% < 2% and the RSDs of RPA were less than 5.64% < 7%, which demonstrated the good precision, reproducibility and stability of methodology. Table S1 summarized the data.

**Table S1** Injection precision, reproducibility and stability for fingerprint analysis

| Peak no. | RSDs of RRT (%) (n = 5) | | |  | RSDs of RPA (%) (n = 5) | | |
| --- | --- | --- | --- | --- | --- | --- | --- |
|  | Precision | Reproducibility | Stability |  | Precision | Reproducibility | Stability |
| 1 | 0.13 | 0.20 | 0.14 |  | 5.01 | 4.02 | 5.64 |
| 2 | 0.21 | 0.11 | 0.23 |  | 2.35 | 2.71 | 3.28 |
| 3 | 0.17 | 0.19 | 0.31 |  | 4.27 | 1.99 | 2.77 |
| 4 | 0.14 | 0.12 | 0.24 |  | 2.83 | 3.95 | 4.19 |
| 5 | 0.19 | 0.22 | 0.18 |  | 1.84 | 2.32 | 2.95 |
| 6 | 0.17 | 0.18 | 0.22 |  | 1.79 | 2.51 | 2.18 |
| 7 | 0.18 | 0.15 | 0.37 |  | 1.71 | 1.87 | 2.47 |

The fingerprints of different batches of LHQW including 3 inferior samples homemade and 40 commercial samples were then established (**Figure S2**) using the professional software "Similarity Evaluation System for Chromatographic Fingerprint of Traditional Chinese Medicine" (Version 2012A, SES software) for evaluating the similarities between different samples. The reference chromatogram was generated with average data.


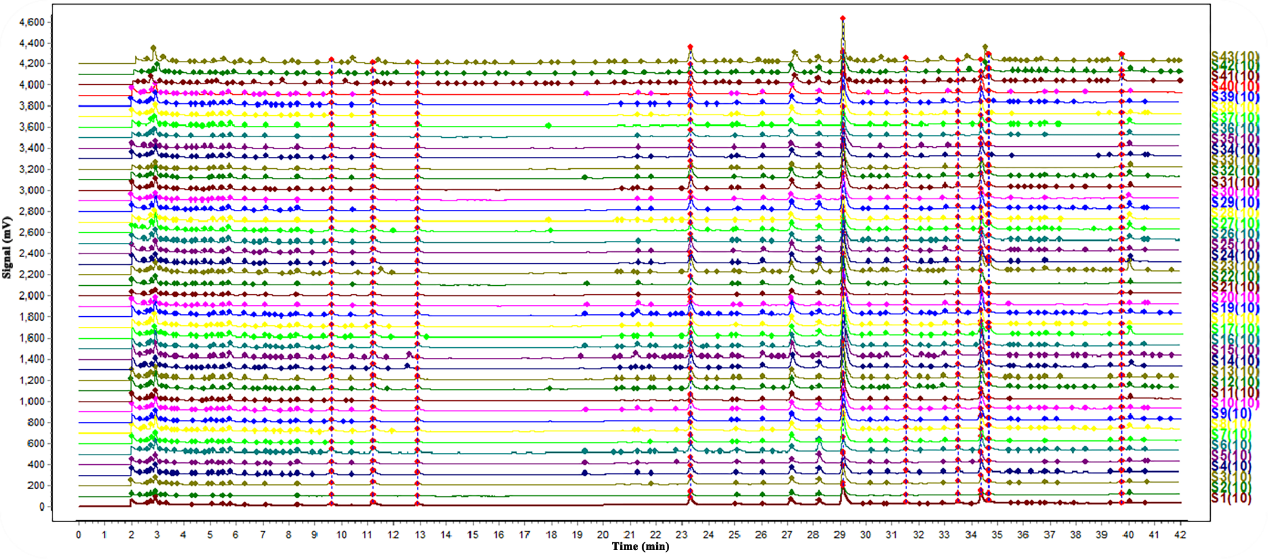
 **Figure S2** Chemical fingerprints of LHQW were used to evaluate the similarities between different samples (S1-S43).

**Table S2** Content determination of seven active ingredient in LHQW capsule

| Samples | | Batch no. | Chlorogenic acid（mg/g） | | Rutin（mg/g） | Forsythiaside A（mg/g） | Phillyrin（mg/g） | Isochlorogenic acid C（mg/g） | Isochlorogenic acid B（mg/g） | | Caffeic acid（mg/g） |
| --- | --- | --- | --- | --- | --- | --- | --- | --- | --- | --- | --- |
| S01 | 1508114 | | 2.43 | 0.69 | 2.04 | 1.45 | 0.81 | 5.50 | 0.99 | |  |
| S02 | 1508117 | | 1.24 | 0.57 | 1.68 | 1.86 | 1.65 | 7.37 | 0.80 | |  |
| S03 | 1508118 | | 1.88 | 0.58 | 1.57 | 1.50 | 0.75 | 3.44 | 0.85 | |  |
| S04 | 1508123 | | 2.32 | 0.67 | 1.47 | 1.45 | 1.03 | 9.10 | 1.09 | |  |
| S05 | 1508129 | | 0.92 | 0.67 | 0.96 | 1.59 | 0.72 | 3.59 | 0.96 | |  |
| S06 | 1508132 | | 1.31 | 0.63 | 1.88 | 1.49 | 0.73 | 5.30 | 1.17 | |  |
| S07 | 1509081 | | 2.65 | 0.75 | 1.92 | 2.16 | 1.86 | 5.94 | 1.02 | |  |
| S08 | 1509084 | | 2.86 | 0.68 | 0.98 | 1.20 | 1.93 | 10.01 | 1.38 | |  |
| S09 | 1510002 | | 1.62 | 0.80 | 1.87 | 2.06 | 0.78 | 5.01 | 1.24 | |  |
| S10 | 1510004 | | 1.59 | 0.84 | 1.58 | 2.11 | 1.58 | 6.93 | 1.31 | |  |
| S11 | 1510017 | | 1.74 | 0.76 | 1.94 | 2.38 | 1.31 | 6.94 | 1.16 | |  |
| S12 | 1510021 | | 2.69 | 0.97 | 1.38 | 2.08 | 1.84 | 8.37 | 1.32 | |  |
| S13 | 1510024 | | 2.42 | 0.87 | 1.55 | 2.35 | 1.21 | 5.97 | 1.15 | |  |
| S14 | 1510028 | | 2.95 | 1.22 | 1.39 | 2.95 | 1.61 | 9.53 | 1.34 | |  |
| S15 | 1510029 | | 2.85 | 1.05 | 1.35 | 3.28 | 1.09 | 7.91 | 1.34 | |  |
| S16 | 1510032 | | 1.07 | 1.01 | 0.97 | 3.12 | 1.03 | 4.50 | 1.41 | |  |
| S17 | 1510035 | | 1.87 | 0.97 | 2.22 | 2.23 | 0.94 | 4.18 | 1.12 | |  |
| S18 | 1510038 | | 2.51 | 1.13 | 1.93 | 2.69 | 1.34 | 7.63 | 1.34 | |  |
| S19 | 1510048 | | 2.47 | 1.12 | 1.84 | 3.24 | 1.26 | 6.13 | 1.40 | |  |
| S20 | 1510051 | | 2.51 | 0.76 | 1.54 | 3.01 | 1.29 | 7.50 | 0.99 | |  |
| S21 | 1511008 | | 2.32 | 0.58 | 1.42 | 2.52 | 1.51 | 8.81 | 0.80 | |  |
| S22 | 1511012 | | 1.87 | 0.77 | 1.09 | 2.94 | 1.68 | 8.83 | 1.08 | |  |
| S23 | 1511050 | | 2.33 | 1.84 | 1.11 | 2.49 | 1.24 | 8.73 | 1.22 | |  |
| S24 | 1511161 | | 2.19 | 0.88 | 1.64 | 2.37 | 0.95 | 5.88 | 1.09 | |  |
| S25 | 1511181 | | 2.39 | 0.69 | 2.05 | 2.58 | 1.86 | 7.53 | 1.23 | |  |
| S26 | 1601012 | | 2.31 | 0.75 | 2.77 | 1.72 | 1.96 | 7.22 | 1.28 | |  |
| S27 | 1601016 | | 2.62 | 0.67 | 0.62 | 1.18 | 0.93 | 6.46 | 1.39 | |  |
| S28 | 1601058 | | 1.76 | 0.68 | 1.85 | 1.14 | 0.72 | 3.59 | 1.09 | |  |
| S29 | 1601059 | | 1.91 | 0.68 | 1.08 | 2.41 | 0.94 | 6.10 | 1.15 | |  |
| S30 | 1601060 | | 1.39 | 0.54 | 1.57 | 1.87 | 0.85 | 5.42 | 0.86 | |  |
| S31 | 1601061 | | 1.90 | 0.81 | 3.03 | 2.01 | 0.71 | 5.42 | 1.10 | |  |
| S32 | 1601062 | | 1.77 | 1.79 | 1.87 | 3.04 | 0.99 | 8.29 | 1.08 | |  |
| S33 | 1601063 | | 1.54 | 0.49 | 1.94 | 2.05 | 2.55 | 8.01 | 0.87 | |  |
| S34 | 1601064 | | 2.62 | 0.89 | 1.89 | 1.77 | 1.87 | 10.41 | 1.31 | |  |
| S35 | 1601065 | | 1.48 | 0.58 | 0.82 | 1.87 | 1.55 | 8.89 | 0.89 | |  |
| S36 | 1601066 | | 1.46 | 0.96 | 1.86 | 2.06 | 1.92 | 6.51 | 0.91 | |  |
| S37 | 1601067 | | 1.63 | 0.62 | 1.53 | 1.21 | 0.69 | 4.24 | 0.96 | |  |
| S38 | 1601079 | | 2.01 | 0.61 | 1.30 | 1.69 | 0.91 | 5.99 | 1.01 | |  |
| S39 | 1602001 | | 2.62 | 0.77 | 1.92 | 1.89 | 1.13 | 7.12 | 1.23 | |  |
| S40 | 1602007 | | 2.92 | 1.06 | 2.16 | 2.38 | 1.29 | 6.65 | 1.42 | |  |
| S41  S42  S43 | A1 | | 1.42 | 0.58 | 0.96 | 1.21 | 0.59 | 2.31 | 0.79 | |  |
|  | A2 | | 1.08 | 0.55 | 1.19 | 1.43 | 0.56 | 2.16 | 0.79 | |  |
|  | A3 | | 1.51 | 0.57 | 1.98 | 2.07 | 0.83 | 3.20 | 0.91 | |  |

1. **UPLC-MS analysis**

Metabolite separation was conducted on Waters Xevo G2-XS QTOF/MS (Waters, Manchester, UK). Chromatography was performed on an Acquity UPLC HSS T3 C18 column (2.1 mm i.d. × 100 mm, 1.8 mm). The column was maintained at 40°C, and subsequently, the mobile phase was composed of 0.1% formic acid in acetonitrile (solvent A) and 0.1% formic acid in water (solvent B) with a linear gradient elution as table S3. The flow rate was 0.30 mL·min^-1^, and 2 μL aliquot of each sample was injected into the column. The eluent was introduced to the mass spectrometry directly without split. The high resolution QTOF mass spectrometer was operated in both positive and negative ion mode using electrospray ionization (ESI). Data were collected in the full scan mode from m/z 50 to m /z 1200. The optimal conditions of analysis were as follows: Collision gas were nitrogen and argon; nitrogen was used as the dry gas, the desolvation gas flow rate was set at 500 L/h, and the cone gas flow was maintained at 50 L/ h; the capillary voltage and the cone voltage were set at 2500 V and 40 V respectively; the desolvation gas flow rate was 800 L/h at a temperature of 350℃; the source temperature was at 110 C. Leucine enkephalin was used as the reference compound (positive ion mode ([M + H]^+^ = 556.2771) and [M - H]^-^ = 554.2615) at a concentration of 0.2 ng /mL under a flow rate of 100 mL/min. LockSpray frequency was set at 10 s and averaged over 10 scans for correction. After every 10 sample injections, a pooled sample followed by a blank was injected in order to ensure consistent performance of the system.

**Table S3** High performance gradient elution conditions

| Time (min) | Acetonitrile (A%) | Waster (B%) |
| --- | --- | --- |
| Initial | 5 | 95 |
| 1 | 5 | 95 |
| 9 | 40 | 60 |
| 19 | 90 | 10 |
| 21 | 100 | 0 |
| 25 | 100 | 0 |

**Table S4** Parameters of PCA and OPLS-DA model

| No. | Model type | Groups included | R^2^X | R^2^Y (cum) | Q^2^Y (cum) |
| --- | --- | --- | --- | --- | --- |
| Data of ESI+ mode | | | | | |
| 1 | PCA | Normal, Model & LHQW | 0.379 | — | 0.475 |
| 2 | OPLS-DA | Normal & Model | — | 0.982 | 0.818 |
| 3 | OPLS-DA | Model & LHQW | — | 0.995 | 0.762 |
| Data of ESI- mode | | | | | |
| 4 | PCA | Normal, Model & LHQW | 0.457 | — | 0.729 |
| 5 | OPLS-DA | Model & Normal | — | 0.986 | 0.793 |
| 6 | OPLS-DA | LHQW & Model | — | 0.989 | 0.810 |

**Table S5** Identification biomarkers detected by LC-Q-TOF-MS in both positive and negative ionization modes

| Mode | Number | t_R_ (min) | Compound | Mass (Neutral) | Formulate |
| --- | --- | --- | --- | --- | --- |
| ESI（-） | 1 | 5.25 | L-Ornithine | 132.0899 | C_5_H_12_N_2_O_2_ |
|  | 2 | 11.59 | Prostaglandin F2alpha | 354.2406 | C_20_H_34_O_5_ |
|  | 3 | 14.84 | Arachidonic acid | 304.24023 | C_20_H_32_O_2_ |
| ESI（+） | 4 | 1.2 | L-Tyrosine | 181.0739 | C_9_H_11_NO_3_ |
|  | 5 | 0.81 | L-Valine | 117.079 | C_5_H_11_NO_2_ |
|  | 6 | 14.2 | Taurocholic acid | 515.2917 | C_26_H_45_NO_7_S |

**Table S6** Result of pathway analysis with MetaboAnalyst 3.0

| Pathway name | Total | Hits | Raw p | -log(p) | FDR | Impact |
| --- | --- | --- | --- | --- | --- | --- |
| Arachidonic acid metabolism | 36 | 2 | 0.0088 | 4.7294 | 0.4605 | 0.3260 |
| Ubiquinone and other terpenoid-quinone biosynthesis | 3 | 1 | 0.0126 | 4.3695 | 0.4605 | 0 |
| Phenylalanine, tyrosine and tryptophan biosynthesis | 4 | 1 | 0.0168 | 4.0835 | 0.4605 | 0.5 |
| Aminoacyl-tRNA biosynthesis | 69 | 2 | 0.0308 | 3.4778 | 0.5360 | 0 |
| Taurine and hypotaurine metabolism | 8 | 1 | 0.0334 | 3.3975 | 0.5360 | 0 |
| Phenylalanine metabolism | 11 | 1 | 0.0457 | 3.0843 | 0.5360 | 0 |
| Valine, leucine and isoleucine biosynthesis | 11 | 1 | 0.0457 | 3.0843 | 0.5360 | 0.3333 |


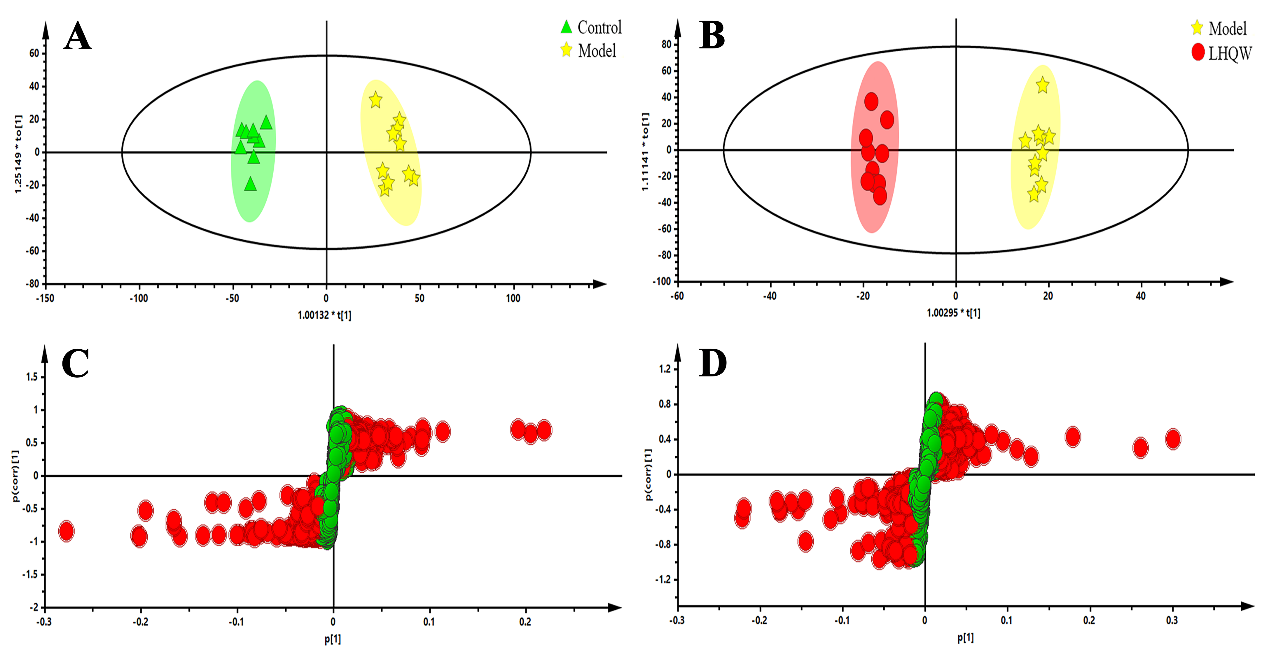


**Figure 3S** OPLS-DA score plots and S-plots generated from the OPLS-DA of the QTOF/MS data from Control, Model and LHQW groups in the ESI+ mode. OPLS-DA score plots were the pair-wise comparisons between the Control and the Model (A) as well as the Model and LHQW (B). S-plot of the OPLS-DA model were for the the Control and the Model (C) and the Model and LHQW (D), whose axes plotted in the S-plot from the predictive component are p1 vs. p(corr)1, representing the magnitude (modeled covariation) and reliability (modeled correlation) respectively.
